# Replication stress by MMS stimulates DNA synthesis in post-replicative G2-phase in *S. pombe* .

**DOI:** 10.17912/micropub.biology.000852

**Published:** 2023-07-06

**Authors:** Seong Min Kim, Susan L. Forsburg

**Affiliations:** 1 University of Southern California, Los Angeles, California, United States

## Abstract

DNA replication is generally limited to S-phase but replication stress can drive cells to undergo DNA synthesis outside of S-phase. Mitotic DNA synthesis pathway is known to be activated to deal with replication stress-induced chromosomal instability. There is also growing evidence that residual DNA synthesis can occur in G2. We demonstrate that fission yeast cells stimulate DNA synthesis in G2-phase but not in M-phase in response to DNA alkylating agent MMS. Auxin-induced degradation of DNA replication helicase Mcm4 during G2, but not during mitosis, inhibits post-replicative DNA synthesis.

**Figure 1. MMS increases DNA synthesis during G2.**
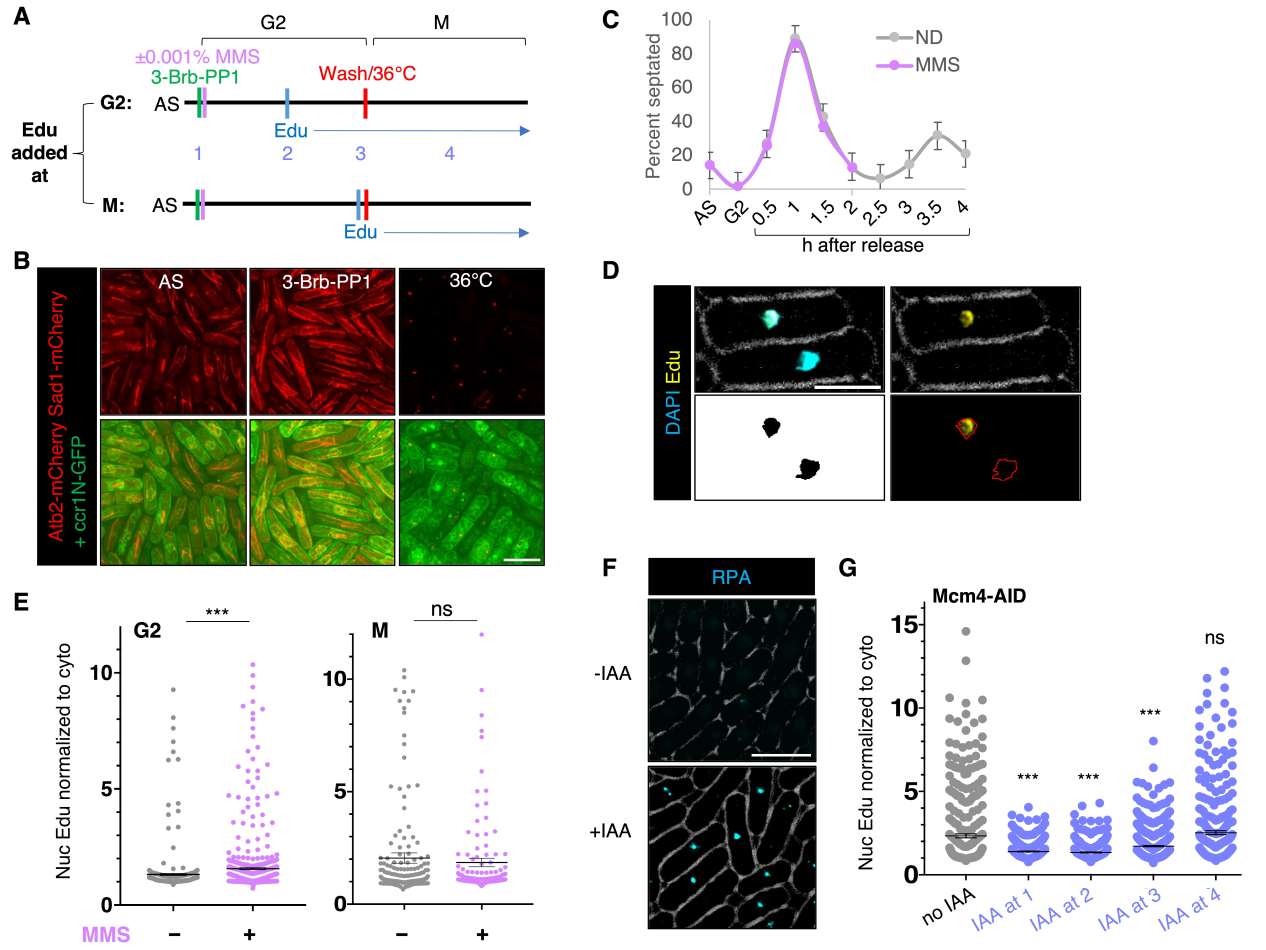
(A) Experimental procedure outline as detailed in Method section. 5-Ethynyl-2’-deoxyuridine (Edu) was added to *cdc2-asM17 cut9-665* cells during G2 arrest (G2) by 3-Brb-PP1 or just before release to 36 ºC for M arrest (M) in the presence or absence of 0.001% MMS. AS indicates asynchronous culture. Numbers 1-4 indicate when auxin (IAA) was added for panel (G). (B) Images of spindle fiber (Atb2-mCherry), spindle pole body (Sad1-mCherry) (top) and nuclear membrane marker Ccr1N-GFP (ccr1(275-678)-GFP) (bottom) in asynchronous culture (AS), after G2-arrest by 3-Brb-PP1, or after M-arrest via restrictive temperature (36°C). Scale bar: 10 µm. (C) Percent of septated cells were quantified after release from G2 arrest by 3-Brb-PP1 in the absence (ND) or presence of 0.001% MMS. (D) Image analysis steps for Edu intensity. Nuclear area defined by DAPI staining was used to assess Edu intensity which was then normalized to cytoplasmic area within the same cell. Scale bar: 5 µm. (E) Quantification of normalized nuclear Edu intensity in G2 or M arrested with or without MMS treatment. (F) Images of RPA-CFP foci in *mcm4-AID* cells with or without IAA. Scale bar: 10 µm. (G) Quantification of normalized nuclear Edu intensity in G2-arrested *mcm4-AID cdc2-asM17 cut9-665* cells. IAA was added at the beginning of G2-arrest (1) , during G2-arrest (2), at the beginning of M-arrest (3), or during M-arrest (4). A two-tailed Student’s t-test was used to determine significance: * P < 0.05, ** P < 0.01, *** P < 0.001, n.s. not significant. Error bars represent Standard Error (SE).

## Description

Replication stress has been proposed to drive DNA synthesis during mitosis (MiDAS) in mammalian cells (Minocherhomji et al. 2015; Bhowmick et al. 2016; Lezaja et al. 2021; Wu et al. 2023). Although the precise mechanism of MiDAS still remains to be defined, several key proteins involved in the process of replisome maintenance (Sonneville et al. 2019), replication structure processing (Minocherhomji et al. 2015; Calzetta et al. 2020; Garribba et al. 2020), and DNA damage repair (Minocherhomji et al. 2015; Bhowmick et al. 2016; Wu et al. 2023) are required for MiDAS. DNA synthesis outside of S-phase does not seem to be limited to mitotic phase as cells exposed to mild replication stress were shown to be able to continue DNA synthesis throughout G2 phase (Mocanu et al. 2022). Studies done in budding yeast (Ivanova et al. 2020) and *C. elegans* (Sonneville et al. 2019) demonstrate that DNA synthesis outside of S-phase also occurs in lower eukaryotes as well.

Replication and cell cycle proteins in *Schizosaccharomyces pombe* , or fission yeast, share homology with human proteins, making it an ideal model system to elucidate the effect of replication stress on genome integrity and checkpoint (rev. in (Vyas et al. 2021)). Low levels of DNA synthesis in post-replicative G2 has been detected in *S. pombe* (Kelly and Callegari 2019). In this study, we investigated whether replication stress can promote DNA synthesis outside of S-phase in fission yeast and which processes and proteins contribute to it.

We used analogue-sensitive *cdc2-asM17* allele to arrest cells in G2 with ATP analog 3-Brb-PP1 ( (3-[(3-bromophenyl)methyl]-1-(1,1-dimethy- lethyl)-1H-pyrazolo[3,4-d]pyrimidin-4-amine) (Aoi et al. 2014; Singh et al. 2021) along with the *cut9-665* temperature-sensitive allele that arrests cells in mitosis (Samejima and Yanagida 1994). The strains were engineered to take up the thymidine analogue Edu (5-Ethynyl-2’-deoxyuridine) (Hodson et al. 2003), which was added in the middle of G2 arrest or just before mitotic arrest to observe Edu uptake in G2 or M ( Figure 1A, Table 1). To confirm that G2-arrest induced by 3-Brb-PP1, we imaged for spindle fiber (Atb2-mCherry) ( Figure 1B ). 3-Brb-PP1 treatment induced uniformly extended fibers along the long axis of the cell as typically found during interphase, whereas asynchronous (AS) culture had a mixture of extended fibers and short and thick mitotic spindles. Placing cells at the restrictive temperature (36°C) to arrest cells at mitosis via the *cut9-665* allele ablated spindle fibers, leaving only the spindle pole body (Sad1-mCherry) visible. Replication stress was induced by 0.001% MMS, at a concentration that did not perturb cell cycle ( Figure 1C ). After Click-it reaction, Edu signal was visualized alongside DAPI (4’,6-diamidino-2-phenylindole) staining ( Figure 1D ). Nuclear Edu signal intensity was then normalized to cytoplasmic signal for quantification ( Figure 1D -E).

We compared Edu intensity in untreated cells and MMS-treated cells after Edu was added during G2 or M ( Figure 1A ). MMS induced greater Edu intensity when Edu was added during G2-arrest but not when Edu was added during M-arrest ( Figure 1E ). This suggests that MMS-induced DNA synthesis mainly occurs during G2 not M. To further confirm this, we used auxin-inducible degron system in the DNA replication MCM helicase subunit, Mcm4 (Table 1, (Watson et al. 2021)). In this system, Mcm4 protein is degraded within 15 minute after auxin (5’adamantyl-IAA, IAA) was added (Watson et al. 2021). Auxin-induced Mcm4 degradation also resulted in DNA damage marked with ssDNA binding protein RPA ( Figure 1F ). We reasoned that if DNA synthesis is limited to G2, Mcm4 degradation during G2 will reduce nuclear Edu level but Mcm4 degradation during M would not. Indeed, this was the case as addition of IAA before and during G2-arrest significantly reduced Edu signal ( Figure 1G ). Adding IAA just before M-arrest also reduced Edu level but to a much lesser extent than when IAA was added during G2. Adding IAA during M-arrest had no reduction in Edu level. These results indicate that Mcm4-depedent DNA synthesis is occurring in G2 not M.

In summary, this study confirmed that some DNA synthesis occurs in post-replicative G2 phase in fission yeast and that ongoing replication stress such as chronic MMS exposure induces more DNA synthesis in G2. We have previously showed that replication mutants that are presumed to be arrested in late S-phase lack nuclear accumulation of Tos4 which is normally upregulated during S-phase (Kim et al. 2020). Findings from this study support our previous suggestion that these replication mutants may indeed be arrested in G2 as we demonstrated that cells promote G2 DNA synthesis under replication stress. Attempt and failure to resolve replication stress may cause these replication mutants to be arrested in G2. How DNA replication carries over from S- to G2-phase remains a critical question to answer in future investigations.

## Methods

**Yeast strains and Media**

*Schizosaccharomyces pombe* strains (Table 1) were cultured using standard protocols and media (Sabatinos and Forsburg 2010), grown in supplemented Edinburgh minimal medium (EMM).

Table 1.

**Table:** 

Strain	Genotype	Source
FY10617	h- cut9-665 cdc2-asM17 leu1-32::hENT1-leu1+(pJAH29) his7-366::hsv-tk-his7+(pJAH31) ura4-D18	(Singh et al. 2021)
FY10739	h+ mcm4-aid-V5-Turg1:kanMX6, arg3::bleMX6-arg3+-Padh1-OsTIR1(F74A)-TADH1 cut9-665 cdc2-asM17 leu1-32::hENT1-leu1+(pJAH29) his7-366::hsv-tk-his7+(pJAH31)	(Watson et al. 2021)

**Edu uptake assay**

Cells grown in asynchronous culture were arrested in G2 by 2 µM 3-Brb-PP1 (TRC, A602985) for 3.5 h with or without 0.001% of methyl methanesulfonate (MMS) and then were washed twice with supplemented media before being placed at 36 ºC for M-arrest. 10 µM 5-Ethynyl-2’-deoxyuridine was added either during G2-arrest or before M-arrest. 100 nM 5’adamantyl-IAA (TCI Chemicals, A3390) was added at or during G2- or M-arrest. Cells were spun down and resuspended in 70% ethanol and placed in 4 ºC for fixation. Then fixed cells were washed in 1% BSA containing PBS and processed using Click-iT™ EdU Cell Proliferation Kit for Imaging, Alexa Fluor™ 488 dye (ThermoFisher Scientific, cat #10337), following the manufacturer protocol.

**Microscopy**

For live cell imaging, cells were placed on 2% agarose pads sealed with VaLaP (1/1/1 [wt/wt/wt] Vaseline/lanolin/paraffin). Click-iT processed samples were suspended in 20 µl of 1% BSA and then transferred to charged slides (Premiere, 9308W) and heat-fixed at 50 ºC for 5 min. Antifade mounting medium (50% glycerol in water with 0.1% p-phenylenediamine dihydrochloride) with 1 µg/ml DAPI (4’,6-diamidino-2-phenylindole) was then added before placing the coverslip for imaging. Images were acquired using a DeltaVision microscope (with softWoRx version 4.1; GE, Issaquah, WA) using a 100x (1.35, U Plan Apo, PSF, IX70) lens, solid-state illuminator, and 12-bit CCD camera. Images were deconvolved and maximum intensity projected for fluorescence images (softWoRX) and transmitted light images were inverted and added for outline of the cells (ImageJ) (Schindelin et al. 2012). Snapshot images were projected from seven z-stacks of 0.5 mm with 0.08-0.5 sec exposure time.

**Image analysis**

ImageJ was used to create binary image of the nucleus of each cell from DAPI staining ( Figure 1D ) and this ROI (region of interest) was used for assessing Edu-488 intensity in the nucleus. The same area size was then translated to cytoplasmic part within the cell for normalization. Nuclear Edu-488 intensity over cytoplasmic intensity was plotted using GraphPad. Figure 1E for WT strain shows the data collected from three biological replicates and Figure 1G for MCM4-AID strain shows data from two biological replicates.
